# Supplementary material for: Predictability of fossil fuel CO2 from air quality emissions
Source: Nat Commun. 2023 Mar 23;14:1604. doi: 10.1038/s41467-023-37264-8 (PMC10034258; doi:10.1038/s41467-023-37264-8)
Supplement: Supplementary file 1 — Supplementary Information [file 41467_2023_37264_MOESM1_ESM.pdf]

Supplementary Information for

Predictability of fossil fuel CO2 from air quality emissions

Kazuyuki Miyazaki, Kevin, Bowman

Corresponding Kazuyuki Miyazaki.
E-mail: kazuyuki.miyazaki@jpl.nasa.gov

This PDF file includes:

Figs. S1 to S7

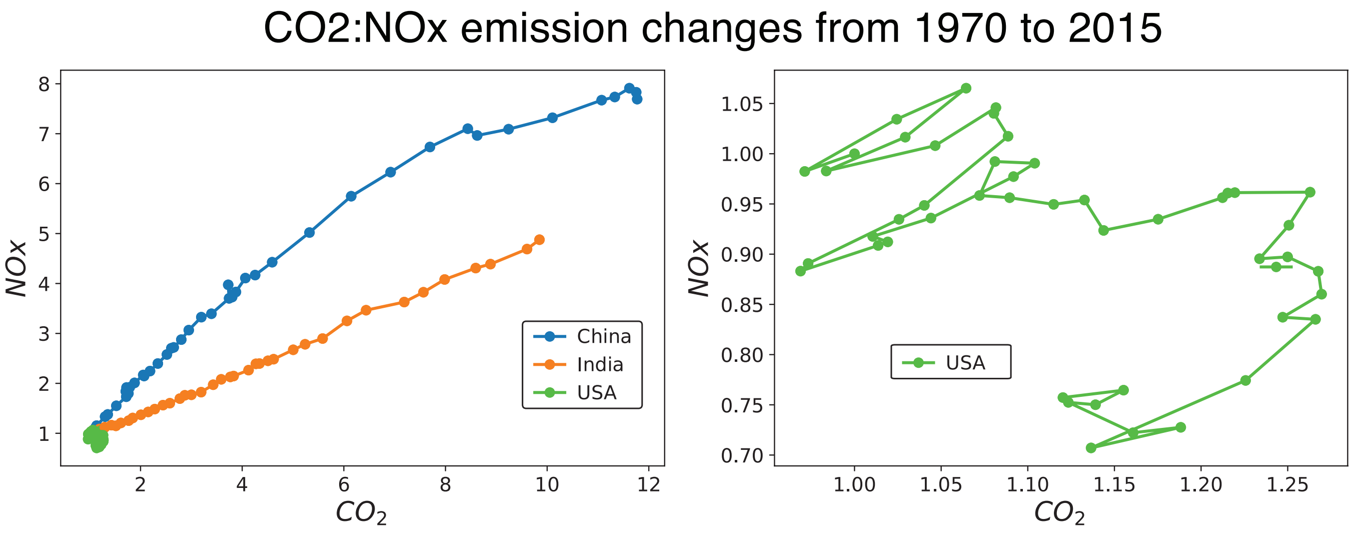

Fig. S1. (left) Co-evolution of country-total anthropogenic emissions of CO_2 (x-axis) and NO_x (y-axis) from 1970 through 2015 from the EDGAR inventories. The values normalized at the 1970 level are shown for China, India, and USA. (right) Same as the left figure, but the result for the USA only are shown.

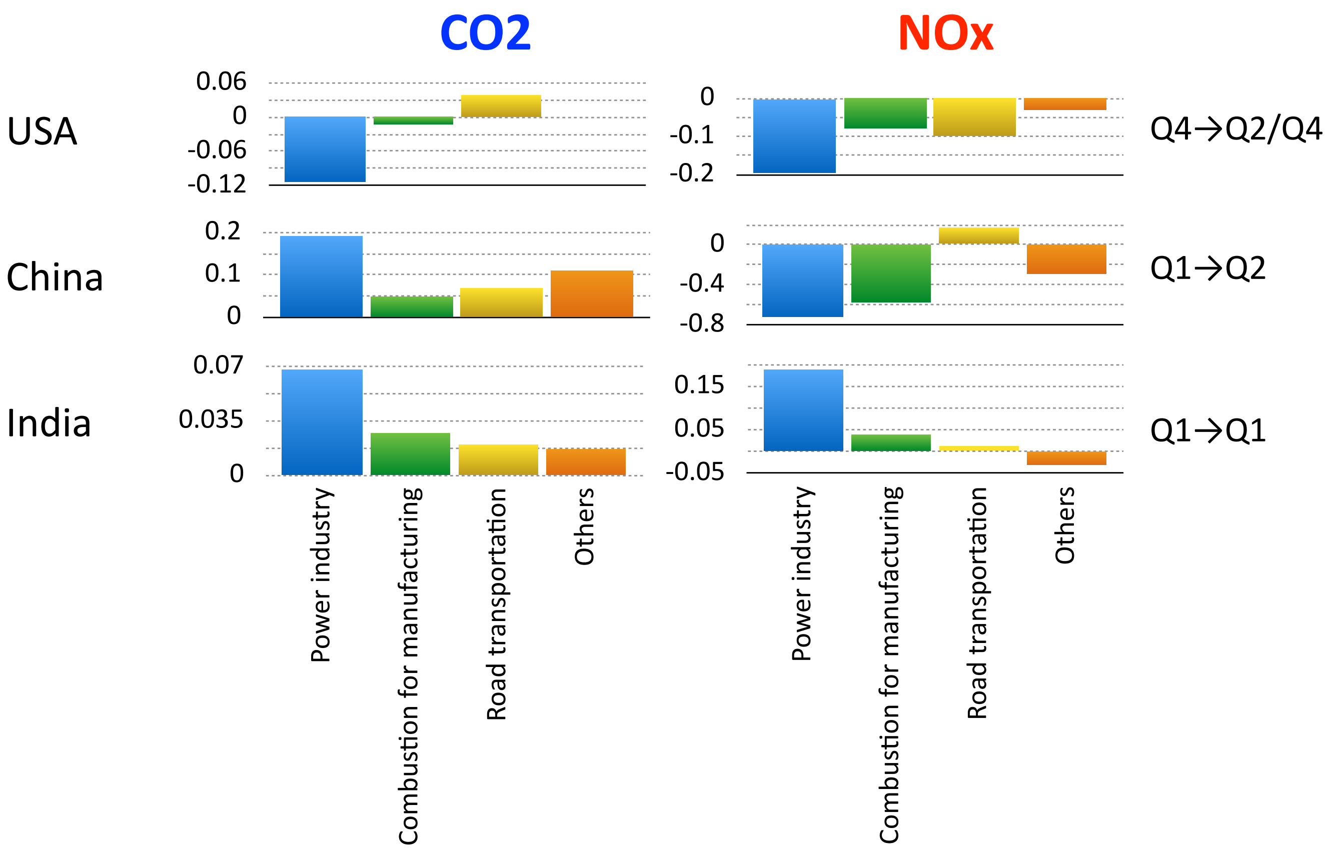

Fig. S2. Changes in annual total emissions from 2011 to 2018 obtained from the EDGAR inventory for each emission sector separately: power industry, combustion for manufacturing, road transportation, and others. The results are shown for China, India, and USA. The unit is PgC for CO_2 (left) and TgN for NO_x (right).

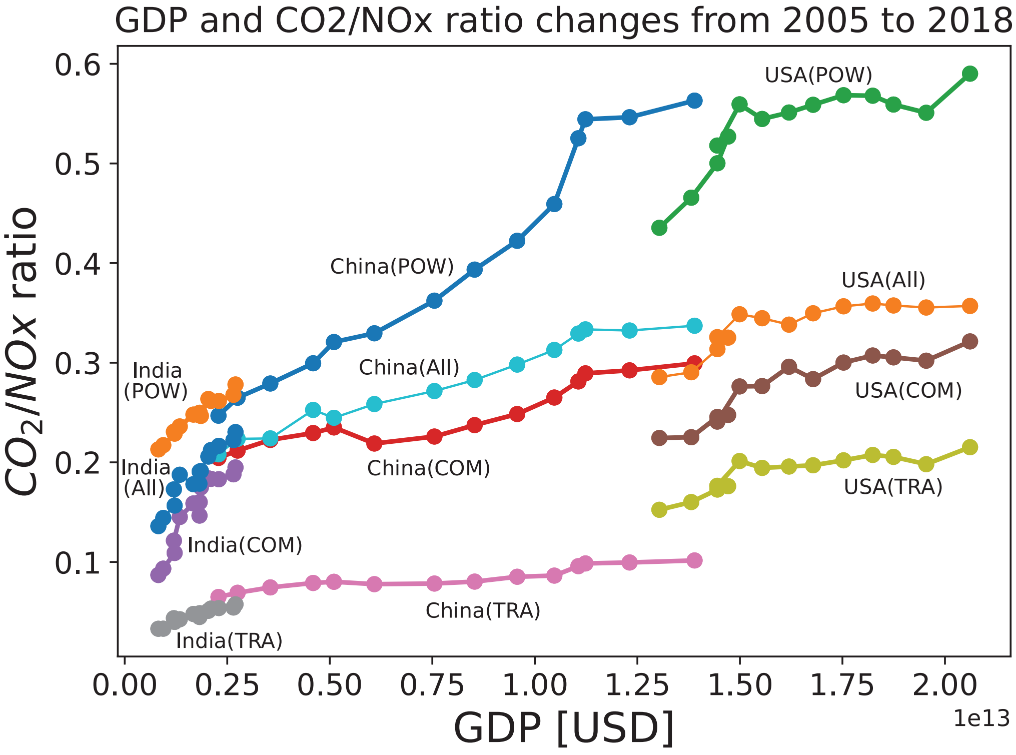

Fig. S3. Relationships between Gross domestic product (GDP, in USD) and CO_2/NOx emission ratio for each emission sector separately: power industry (POW), combustion for manufacturing (COM), road transportation (TRA), and for total emissions (All). The results are shown for China, India, and USA.

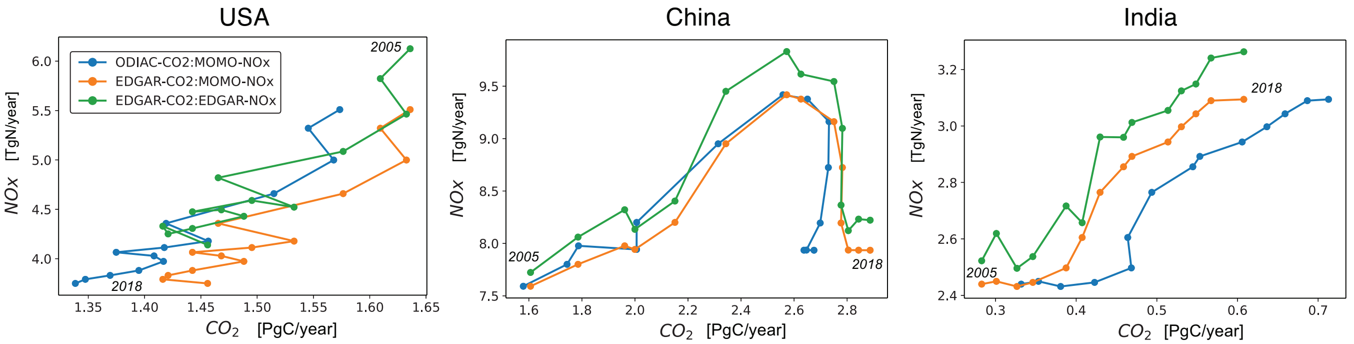

Fig. S4. Co-evolution of CO_2 and NO_x emissions using different emission inventories for USA, China, and India: ODIAC CO_2 and TCR-2 NO_x (blue), EDGAR CO_2 and TCR-2 NO_x (orange), and EDGAR CO_2 and EDAR NO_x (green).

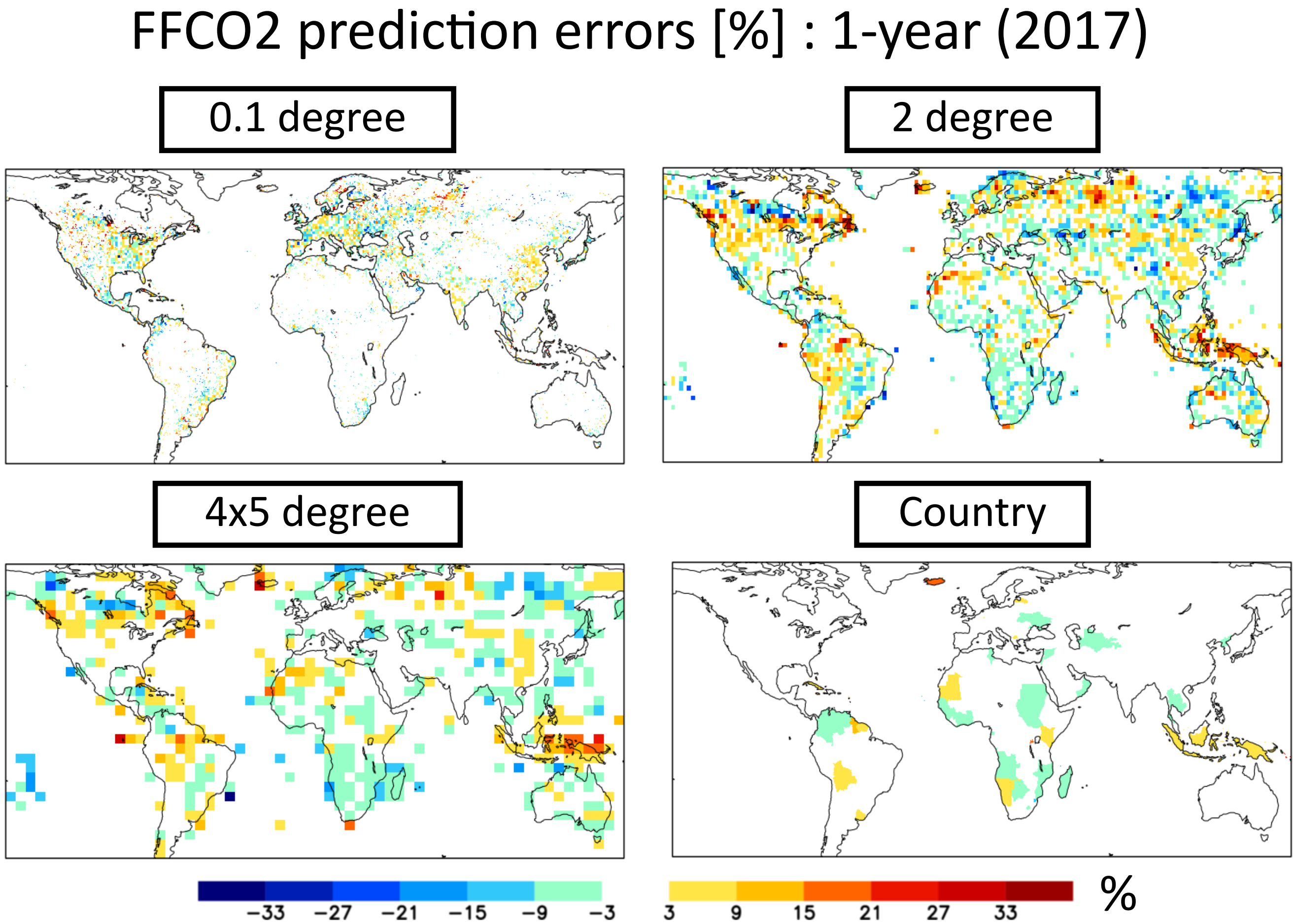

Fig. S5. Spatial maps of 1-year Kalman filter (KF) prediction errors of fossil fuel CO_2 ($FFCO_2$) in 2017 starting from 2016. The results are shown for relative errors (in %) for KF predictions conducted at different spatial resolutions: 0.1x0.1 degree (native inventory resolution), 2x2 degree, 4x4 degree, and country-scale.

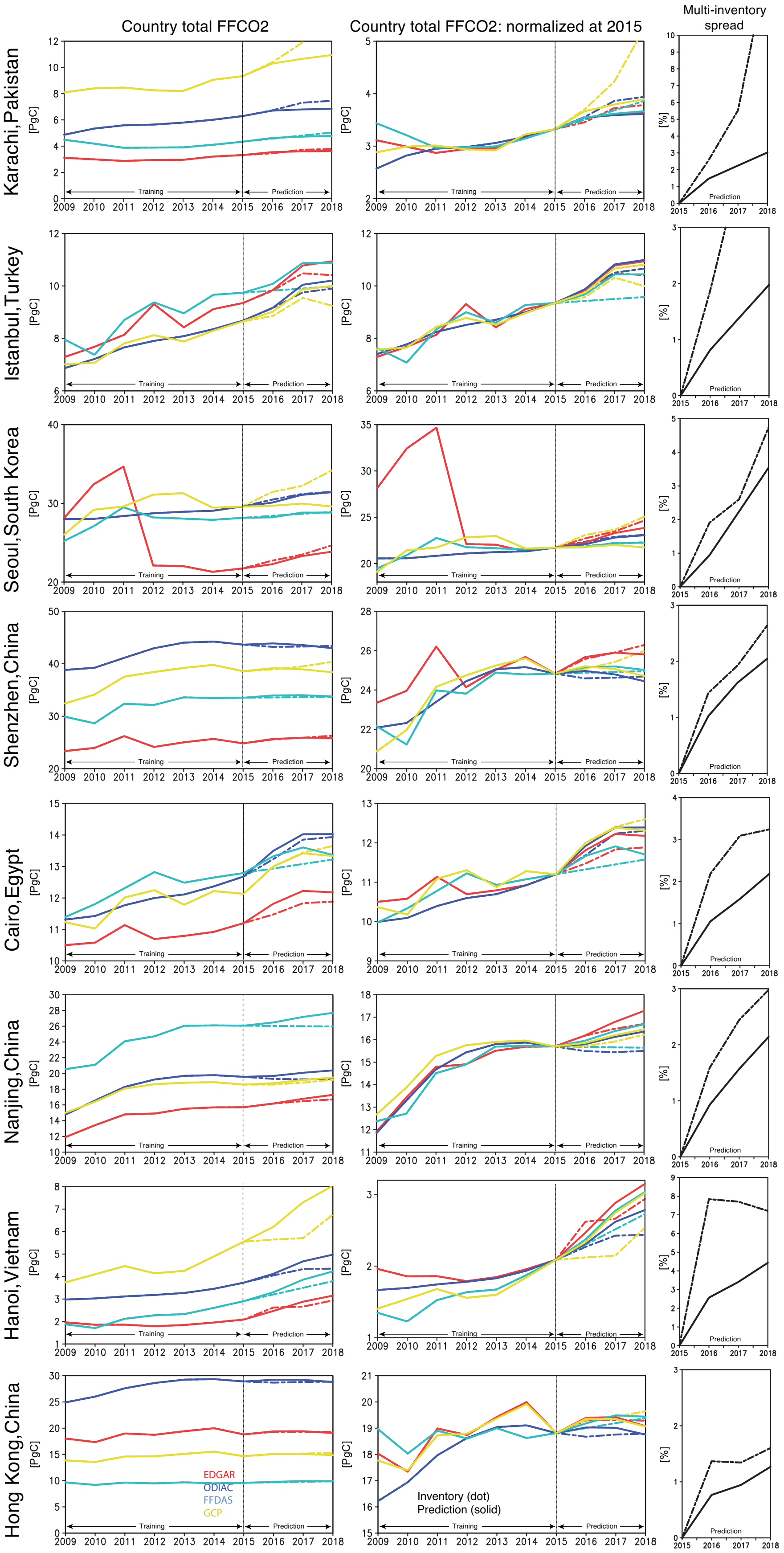

Fig. S6. Same as Fig. 5, but for several selected cities.

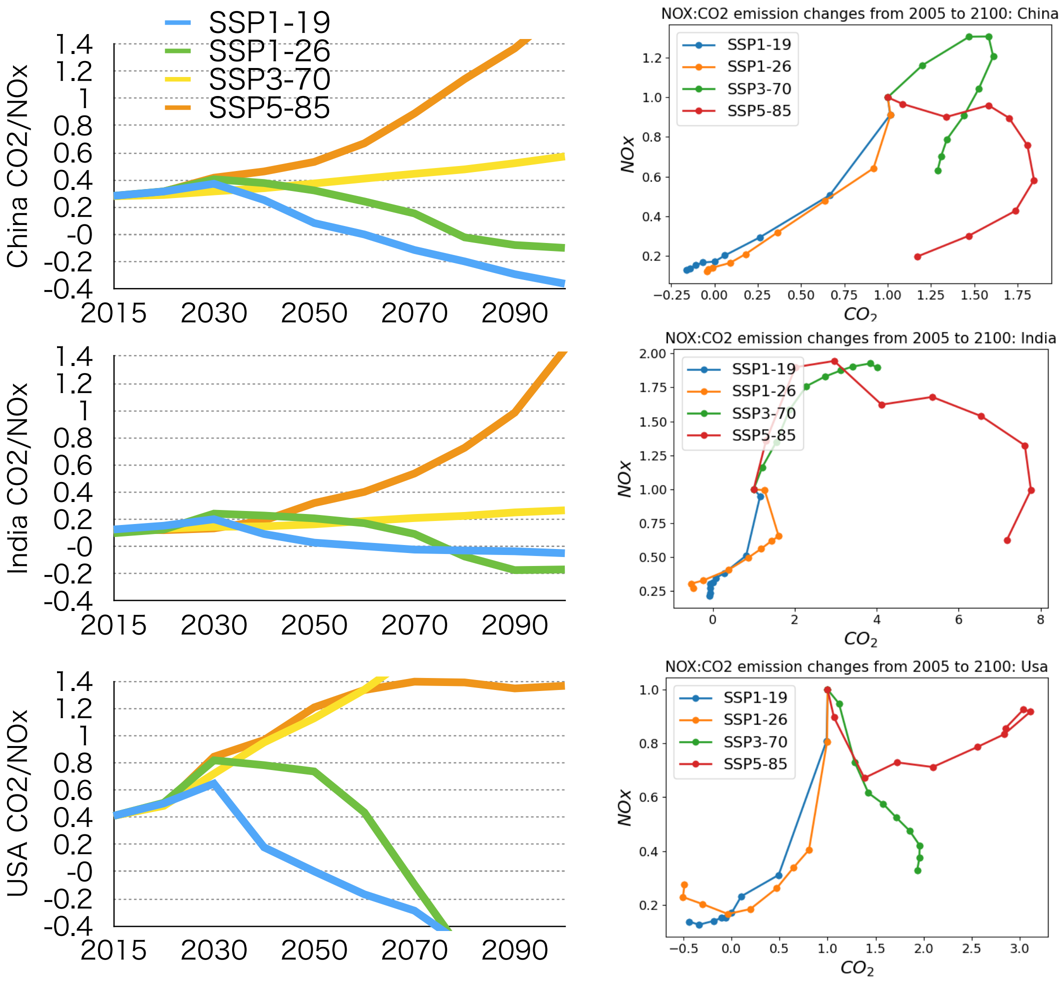

Fig. S7. (left) CO_2/NO_x emission ratio (in PgC/TgN) changes and (right) co-evolution of CO_2 and NO_x emissions normalized at the 2015 values obtained from the Shared Socio-economic Pathways (SSPs) scenarios from 2015 to 2100. Here we compare four SSPs: SSP1-19 (the IPCC's most optimistic scenario, where global CO_2 emissions are cut to net zero around 2050), SSP1-26 (the next-best scenario. Global CO_2 emissions are cut severely, but not as fast, reaching net-zero after 2050), SSP3-70 (emissions and temperatures rise steadily and CO_2 emissions roughly double from current levels by 2100), and SSP5-85 (Current CO_2 emissions levels roughly double by 2050. The global economy grows quickly, but this growth is fueled by exploiting fossil fuels and energy-intensive lifestyles). The results are shown for China, India, and USA.
